# Comparison of Cerebral Micro‐Embolization Detected by Transcranial Doppler Examination Between Radiofrequency Ablation and Pulsed Field Ablation for Atrial Fibrillation

**DOI:** 10.1111/jce.70140

**Published:** 2025-10-13

**Authors:** Tobias Schreiber, Regina von Rennenberg, Tim Bastian Brämswig, Ulf Landmesser, Gerhard Hindricks, Patrick Nagel, Verena Tscholl, Martin Huemer, Johannes Lucas, Philipp Attanasio, Christian H. Nolte

**Affiliations:** ^1^ Deutsches Herzzentrum der Charité, Klinik für Kardiologie Angiologie und Intensivmedizin Berlin Deutschland; ^2^ Klinik für Neurologie, Klinik und Hochschulambulanz für Neurologie Charité‐Universitätsmedizin Berlin, Freie Universität Berlin, Humboldt‐Universität zu Berlin Berlin Germany; ^3^ Center for Stroke Research Charité‐Universitätsmedizin Berlin, Freie Universität Berlin Berlin Germany; ^4^ German Center for Neurodegenerative Diseases (DZNE) Berlin Germany; ^5^ German Centre for Cardiovascular Research (DZHK) Berlin Germany; ^6^ Berlin Institute of Health (BIH) Berlin Germany

**Keywords:** atrial fibrillation, microembolism, pulsed field ablation, radiofrequency ablation

## Abstract

**Background and Aims:**

Pulsed Field Ablation (PFA) can establish pulmonary vein isolation via irreversible electroporation and is believed to reduce overall complications; however, periprocedural stroke has also been reported after PFA procedures. Assessment of microembolic signals (MES) measured by transcranial doppler (TCD) allows to monitor cerebral embolization during the procedure and to identify higher risk procedural steps. This study was designed to compare the incidence of MES during different procedural steps between two different PFA systems and radiofrequency ablation (RFA).

**Methods and Results:**

Pulmonary vein isolation was performed either using either high‐power short‐duration radiofrequency ablation (HPSD RFA), or PFA using a variable loop circular catheter (VLCC) or a circular multielectrode array catheter (CMAC). MES were compared during transseptal puncture, ablation, left atrial mapping, and introduction/retraction of catheters. Neurological examination was performed pre and post ablation for all patients including the National Institutes of Health Stroke Scale (NIHSS).

Fifty‐six consecutive patients (19 female (34%), average age 70 years) were included: 28 in the HPSD RFA group and 14 patients each in the VLCC and CMAC groups. Total MES count was higher for both PFA systems compared to HPSD RFA (VLCC 1402 (IQR 973), CMAC 449 (IQR 193) vs. HPSD RFA 131 (IQR 250); *p* < 0.001). MES count was higher in VLCC than in CMAC (*p* < 0.001). In all groups, MES were most frequently detected during ablation (87% of overall MES).

Postprocedural NIHSS did not differ between groups (median NIHSS 0 [IQR 0] in all groups), yet two patients experienced new focal neurological deficits after PFA.

**Conclusion:**

Pulsed field ablation was associated with a higher cerebral MES count than high‐power short‐duration radiofrequency ablation, with marked differences between catheter systems. Transcranial doppler indicates ablation as the most embolic step in both PFA and high‐power short duration ablation.

## Graphical abstract

Transcranial doppler monitoring of microembolic signals during atrial fibrillation ablation.

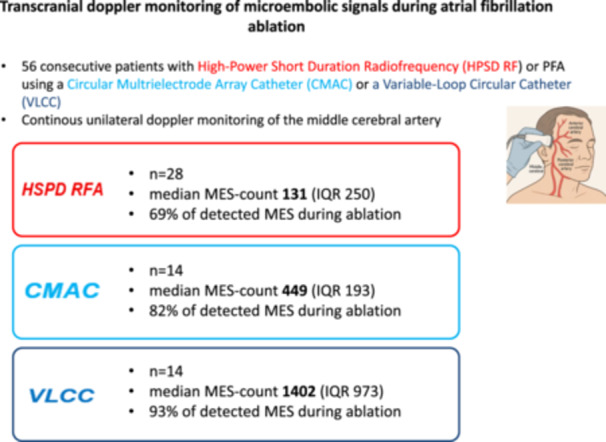

## 1 Introduction

Pulsed field ablation (PFA) is a promising new ablation method establishing pulmonary vein isolation (PVI) via irreversible electroporation. However, safety concerns have emerged, as clinically silent cerebral lesions (SCL) after PFA were seen in 3%–19% of patients [1, 2, 3, 4], and recent reports indicated an increased risk for cerebral ischemia for individual ablation systems [5].

Transcranial doppler (TCD) can be used to detect and quantify microembolic signals (MES) and therefore assess intraprocedural stroke risk [6]. MES burden has been measured during various electrophysiology procedures like cryoballoon [7, 8] and high‐power short‐duration radiofrequency ablation (HPSD RFA) for atrial fibrillation (AF) [9, 10, 11, 12]. So far, data on MES burden during PFA is limited to a single abstract [13].

In this prospective, observational study, all participating patients underwent continuous TCD examination during catheter ablation (CA) for atrial fibrillation to compare MES burden between different ablation systems (HPSD RFA (Thermocool Smarttouch SF catheter (Biosense Webster Inc); VARIPULSE™ variable‐loop circular catheter (VLCC; Biosense Webster Inc.; Irvine, CA) and Circular Multielectrode Array Catheter (CMAC, PulseSelect (Medtronic, Minneapolis, MN, USA)) and to identify procedural steps with an increased risk for cerebral embolization.

## 2 Methods

### 2.1 Study Design and Patients

Patients who underwent first time CA of paroxysmal or persistent AF were included in this prospective, observational study at Deutsches Herzzentrum of Charité – Universitätsmedizin Berlin, between 2024 and 2025. Inclusion criterion was the presence of a transcranial acoustic bone window for MES detection. Patients with previous left atrial thrombi and valvular AF were excluded from the study. The decision to perform either HPSD RFA or PFA was made by the treating physician and was not affected by our study.

The study was approved by the local Ethics Committee of Charité – Universitätsmedizin Berlin (EA1/215/20) and all patients provided written informed consent.

Information on patients' medical history was taken from medical records. This included calculation of the CHADS‐Vasc score; Patients' thrombocyte count and estimated glomerular filtration rate were derived from clinical routine. Patients underwent transthoracic echocardiography as part of clinical routine. Left ventricular ejection fraction and left atrial volume index were derived from the echocardiogram reports.

### 2.2 Ablation Procedure

Left atrial thrombus was ruled out before ablation in patients not under continuous oral anticoagulation or with prior cerebral ischemia via transesophageal echocardiography.

In patients under continuous oral anticoagulation, oral anticoagulation was uninterrupted. The procedure was performed under conscious sedation using a combination of propofol and fentanyl. After ultrasound‐guided cannulation of the right femoral vein, a 10‐pole diagnostic electrophysiological catheter (Inquiry, St Jude Medical, Saint Paul, MN, USA) was placed in the coronary sinus. Heparin was administered before transseptal puncture according to the patient's weight to reach an activated clotting time (ACT) > 300 s for HPSD RFA [14], > 350 s for CMAC [15] and VLCC [16]. ACT was measured every 20 min.

In HPSD RFA and VLCC, a deflectable 8.5 french Agilis sheath (Abbott, Chicago, IL, USA) and a 98 cm BRK‐1 transseptal Needle (St. Jude Medical, St. Paul, MN, USA) was used for transseptal puncture [17]. For CMAC procedures, a 10 french sheath (FlexCath Contour™, Medtronic, Dublin, Ireland) was used according to the manufacturers recommendation [18]. Sheaths were aspirated immediately before transseptal puncture and after introduction of the catheters to avoid air embolism into the left atrium. All sheaths were continuously flushed with saline (2 ml/min) starting before catheter introduction into the venous system. In case of persistent AF, patients were cardioverted before mapping.

In patients undergoing HPSD RFA, electroanatomical mapping (CARTO 3, Biosense Webster Inc) using a pentaspline catheter (Pentaray, Biosense Webster Inc) of the left atrium was performed. For HPSD RFA, pulmonary vein isolation was performed using a Thermocool Smarttouch SF catheter (Biosense Webster Inc). Interlesion distance target was 5 mm, ablation index 550 for the anterior and 400 for the posterior wall and roof with power set at 50 W [19]. Successful isolation was demonstrated by entrance and exit block.

One PFA system used was the PulseSelect System (Medtronic, Minneapolis, MN, USA). The CMAC catheter consists of nine electrodes positioned in a circular shape with an incomplete loop and a diameter of 25 mm [15, 18]. Using a 0.032” J‐tip guidewire, the ablation catheter was placed ostially at the pulmonary vein. Positioning was assessed by fluoroscopy; pulmonary vein angiography was not performed. A minimum of four PFA‐deliveries were applied ostially in each vein, triggered to the ventricular refractory period. One PFA delivery consisted of four biphasic, bipolar energy impulses followed by a 1‐s pause after each impulse [15, 18]. After each energy delivery, the catheter was rotated to enable overlapping lesions. A minimum of four antral ablations were added, and additional ablation pulses were repeated, until full electrical isolation was achieved. Before PFA delivery, phrenic nerve capture was tested to avoid injury and enable catheter repositioning. Electroanatomical mapping was performed after the ablation using a pentaspline catheter (Pentaray, Biosense Webster Inc) [15] to confirm electrical isolation.

The second PFA system used was the VARIPULSE™ variable‐loop circular catheter (VLCC; Biosense Webster Inc.; Irvine, CA) [16, 20]. The VLCC was advanced through an 8.5 french steerable sheath and electroanatomical mapping of the left atrium was performed. Consecutively, at least four ablations per vein (two ostially, two antrally) were performed to achieve full electrical isolation. The TPI (Tissue Proximity Indicator) software on the CARTO 3™ System was used to assess tissue contact; overlapping electrodes were deactivated before ablation. PFA was applied in a bipolar configuration with an energy of 1800 V. Each PFA consists of biphasic pulses, for a total application duration of about 250 ms with 10‐s pauses between trains [20]. The catheter was continuously flushed with an irrigation rate of 4 mL/min [16]. After ablation, electroanatomical mapping was performed using the pentaspline catheter (Pentaray, Biosense Webster Inc).

### 2.3 Transcranial Doppler Examination and MES Detection

**Figure 1:**
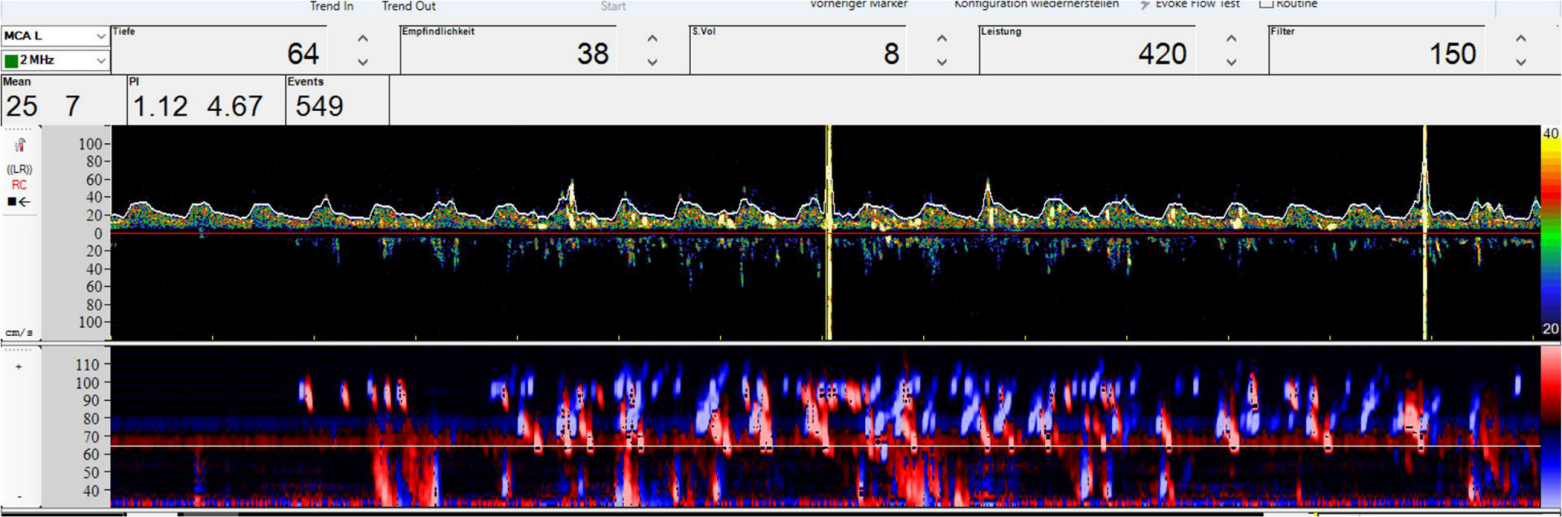
Transcranial doppler ultrasound of the left middle cerebral artery demonstrating the settings including depth, detection threshold, sample volume and high‐pass filter. In both panels (upper panel with spectral Doppler flow velocity waveform, lower panel presenting a color‐coded representation of flow direction and intensity) multiple microembolic signals are visible.

TCD was performed using a DWL Multi‐Dop T2 system (DWL Elektronische Systeme GmbH) and one pulsed‐wave 2‐MHz Doppler probe fixed to the patient's head with the DiaMon (DWL) system. The Doppler probes were used to insonate the middle cerebral arteries at a depth of 50–58 mm with a sample volume of 10 mm. The detection threshold for MES was adjusted to 9 dB. A high‐pass filter was set at 100 Hz (see Figure 1). MES were measured automatically at two depths with a distance of 5 mm. To exclude artifacts, each detected MES was manually controlled by a neurologist after the procedure was completed. As previous studies found no difference between the number of MES in the right and left middle cerebral arteries (7), we examined MES on one side (right or left middle cerebral artery) of each patient depending on better signal quality throughout the procedure. The acoustic signals for MES detection were muted, and the screen displaying the MES was not visible to the ablation team.

MES were assigned to the following procedural steps: (1) venous puncture, coronary sinus catheter placement, transseptal puncture, (2) introduction and positioning of catheters, cardioversion (3) electroanatomical mapping, (4) ablation and (5) removal of the catheters.

### 2.4 Neurological Examination

Clinical examination was performed before and after the procedure (within 24 h) using the National Institutes of Health Stroke Scale (NIHSS). The NIHSS was performed by the same neurologist who performed the TCD measurements.

### 2.5 Statistical Analysis

Since no data on MES and PFA was available, sample size calculation was based on pilot data from the first 10 patients in the HPSD RFA group and five patients each in the CMAC and VLCC groups. Median MES counts were 110.5 (HPSD), 319 (CMAC), and 947 (VLCC). The largest observed pairwise effect (HPSD vs. VLCC) resulted in a Cohen's d > 1.2. For the overall sample size calculation across three groups, this corresponded to a Cohen's f of approximately 0.5. Based on this, a total sample size of 56 patients (28 in HPSD RFA, 14 each in CMAC and VLCC) was used to detect significant group differences (α = 0.05, power = 0.80). An asymmetric allocation was chosen to prioritize statistical power in the HPSD group, which served as the main comparator, while still maintaining adequate group sizes in the CMAC and VLCC arms to allow comparisons.

Data are presented as absolute numbers and percentages for categorical variables or mean ± standard deviation (SD) for continuous variables or median + interquartile range (IQR) for non‐normally distributed continuous variables. Categorical variables were compared using χ2 test. Skewed data was compared using the non‐parametric Kruskal–Wallis test. For normally distributed data, Analysis of Variance (ANOVA) was employed to assess whether there were statistically significant differences between the three ablation groups. In cases where statistical differences were detected, post‐hoc pairwise comparisons were performed using Bonferroni‐correction to control for multiple testing. Analyses were conducted using SPSS software version 22 (SPSS Inc., Chicago, IL, USA). We used a two‐sided significance level of 0.05.

## 3 Results

### 3.1 Patient Characteristics

A total of 28 consecutive patients undergoing HPSD RFA and 28 consecutive patients treated with PFA were included in this study. The PFA group consisted of two subgroups, with 14 patients treated using the CMAC and 14 patients treated with the VLCC. Initially, 68 patients were screened for eligibility. However, 12 patients (18%) were excluded due to the absence of an adequate transcranial ultrasound window. Baseline characteristics are shown in Table 1. The CHADS‐Vasc score and cardiovascular risk factors were evenly distributed amongst the two groups. Previous ischemic stroke or TIA and preexisting neurological deficits were numerically higher in the RFA ablation group.

**Table 1:** Patient characteristics.

	Total (*n* = 56)	HPSD RFA (*n* = 28)	CMAC (*n* = 14)	VLCC (*n* = 14)	*p* value
Age – mean, years (SD)	69.84 (10.36)	71.00 (9.19)	66.21 (13.08)	71.14 (9.40)	0.324
BMI – mean, kg/m^2^ (SD)	27.40 (4.30)	26.44 (3.73)	28.71 (3.76)	27.30 (5.58)	0.231
Gender (female), n (%)	19 (34%)	11 (39%)	2 (14%)	6 (43%)	0.195
CHADS‐Vasc‐Score – median (IQR)	3.00 (2)	3.00 (2)	3.50 (2.25)	2.00 (3)	0.069
Duration of AF – median, months (IQR)	12.00 (28)	14.00 (52)	8.50 (30.25)	11.50 (12.50)	0.660
Previous ischemic stroke or TIA, n (%)	8 (14%)	6 (21%)	1 (7%)	1 (7%)	0.311
Preexisting neurological deficits, n (%)	5 (9%)	4 (14%)	0 (0%)	0 (0%)	0.299
CAD, n (%)	12 (21%)	8 (29%)	3 (21%)	1 (7%)	0.280
Arterial hypertension, n (%)	42 (75%)	23 (82%)	13 (93%)	6 (43%)	0.004
DMT II, n (%)	7 (13%)	2 (7%)	4 (29%)	1 (7%)	0.110
LVEF – median (IQR)	59.50 (8.25)	60.00 (7)	56.00 (21.25)	58.50 (9.75)	0.535
LAVI – mean, ml/m^2^ (SD)	39.89 (13.31)	41.50 (12.85)	44.50 (16.36)	35.33 (12.97)	0.435
Thrombocyte count/nl – mean (SD)	237.80 (64.06)	237.89 (75.52)	240.86 (40.48)	234.50 (62.11)	0.967
eGFR – median, mL/min/1.73 m^2^ (IQR)	70.50 (31.00)	67.50 (27.75)	67.50 (34.50)	76.50 (19)	0.413

Abbreviations: AF, Atrial Fibrillation; BMI, Body Mass Index; CAD, Coronary Artery Disease; DMT II, Diabetes Mellitus Type II; eGFR, Estimated Glomerular Filtration Rate; LAVI, Left Atrial Volume Index; LVEF, Left ventricular ejection fraction.

### 3.2 Procedural Characteristics

Pulmonary vein isolation was acutely successful in all patients, defined as entrance‐ and exitblock in the pulmonary veins. In 13 (48%) patients with RFA, additional ablation of the cavotricuspid isthmus (CTI) was performed after pulmonary vein isolation. MES detected during CTI ablation were excluded from further analysis.

Total procedure duration was shortest in the VLCC group; the dose of heparin administered and ACT were similar in both groups (see Table 2).

**Table 2:** Ablation and procedural characteristics.

	Total (*n* = 56)	HPSD RFA (*n* = 28)	CMAC (*n* = 14)	VLCC (*n* = 14)	*p* value
Persistent AF, *n* (%)	28 (50%)	11 (39%)	11 (79%)	6 (43%)	0.046
Intraprocedural cardioversion, *n* (%)	32 (57%)	12 (43%)	12 (86%)	8 (57%)	0.030
Procedure duration (skin to skin) – min, median (IQR)	67.00 (38)	73.00 (50)	65.50 (30)	54.50 (31)	0.013
Dose of heparin administered – median, U (IQR)	9000.00 (3875)	9000.00 (6000)	9000.00 (6000)	10000.00 (4000)	0.087
ACT, mean (SD)	316.87 (37.37)	308.63 (39.09)	324.38 (36.41)	326.93 (33.03)	0.243
No previous OAC treatment, *n* (%)	5 (9%)	2 (7%)	1 (7%)	2 (14%)	0.719
Left atrial low voltage area > 35%, *n* (%)	10 (18%)	6 (21%)	3 (21%)	1 (7%)	0.518
Additional right atrial ablation (CTI), *n* (%)	13 (23%)	13 (48%)	0	0	< 0.001
Major complications, *n* (%)	3 (5%)	0 (0%)	2 (14%)	1 (7%)	0.144
Postprocedural stroke, *n* (%)	2 (4%)	0	1 (7%)	1 (7%)	0.355

Abbreviations: ACT, Activated Clotting Time; OAC, Oral Anticoagulation.

In the CMAC group, more patients presented with persistent AF than in the two other groups (see Table 2). As the rate of patients with persistent AF was higher in the CMAC group, initial presence of sinus rhythm was lower and intraprocedural cardioversion was performed more frequently (see Table 2). Since cardioversion was performed immediately after catheter introduction, the detected MES during cardioversion were assigned to the second step of our analysis.

Despite the numerically higher number of persistent AF patients in the CMAC group, the number of patients with relevant low voltage areas was similar in all three groups (see Table 2). In the CMAC group, the number of ablation applications did not differ between patients with paroxysmal (34.7 ± 1.5) and persistent AF (39.9 ± 4.8; *p* = 0.094).

Overall, three complications were observed. Two in the CMAC group and one in the VLCC group. In the CMAC group one transient phrenic nerve paralysis occurred, which resolved spontaneously at the end of the procedure. Another patient reported non‐lateralized blurry vision 1 day after the ablation. MRI showed a small infarction affecting the right posterior cerebral artery territory, potentially being the cause for these symptoms. Vision normalized spontaneously within 1 week after ablation. One patient in the VLCC group reported numbness of the left hand, MRI showed acute small infarctions affecting the left middle cerebral artery territory, right posterior cerebral artery territory and right posterior inferior cerebellar artery territory. Symptoms persisted but did not affect the patient's activities of daily living.

### 3.3 Transcranial Doppler Examination, Neurological Examination

In total, 32 271 MES were detected in 56 patients using a unilateral window. Overall, the median MES count per procedure was 387.5 (IQR 309.75). By far the largest number of MES occurred during the procedural step of ablation (*n* = 27 973, 87%), followed by mapping (*n* = 2525, 8%), transseptal puncture (*n* = 935, 3%), catheter introduction (*n* = 782, 2%) and catheter retraction (*n* = 56, 0.002%).

**Figure 2:**
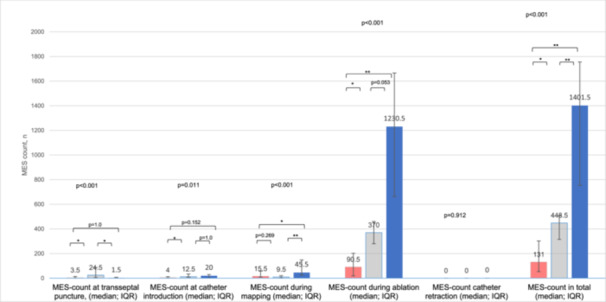
Mean numbers of MES during the different steps of PVI compared between HPSD RFA (red), CMAC (gray) and VLCC (blue). HPSD RFA = high‐power short‐duration radiofrequency ablation, MES = micro‐embolic signals, * indicates a *p*‐value < 0.05, ** indicates a *p*‐value < 0.001.

Figure 2 depicts the number of MES during the five procedural steps for the RFA, CMAC and VLCC group. Total MES count was higher in the PFA groups compared to RFA (CMAC: 449 (IQR 193) vs. HPSD RFA: 131 (IQR 250); *p* < 0.001; VLCC: 1402 (IQR 973) vs. HPSD RFA; *p* < 0.001). A significant difference in MES formation between the two PFA systems was observed as well with a higher number of MES in the VLCC group (VLCC 1402 (IQR 973) vs. CMAC 449 (IQR 193), *p* < 0.001).

For both PFA groups, MES count was significantly higher during ablation. During ablation, MES count for CMAC was increased 4.1 times compared to HPSD RFA; for VLCC, we observed a 13.6‐fold increase (see Figure 2). In the CMAC group, MES count was also significantly higher during transseptal puncture and catheter introduction than in the HPSD RFA group (see Figure 2).

The VLCC and CMAC group did not consistently reach the recommended ACT values of over 350 s (see Table 2). However, a comparison between cases with ACT values above and below 350 s revealed no significant difference in the occurrence of MES (*p* = 0.329).

In the two patients with post interventional neurologic symptoms, MES count was not higher than the average of all PFA ablations (*n* = 448 in the patient treated with CMAC and *n* = 829 in the patient treated with VLCC). Post‐procedural NIHSS was 0 in all but five patients. These five patients had a NIHSS score of 1. Of these, four patients reported that the neurological deficit had been pre‐existing. There were no significant changes between pre‐ and postprocedural NIHSS between gropus.

## 4 Discussion

Patients undergoing CA are at risk for cerebral embolization. Ensuring lowest possible risk for the patient by preventing periprocedural cerebrovascular events remains a key clinical priority, as even subclinical cerebral embolism may contribute to long‐term cognitive decline and increase the risk of stroke [21]. Our findings add to the body of evidence on the cerebral safety profile of CA using pulsed field energy and highlight procedural steps associated with subclinical embolic events.

The three main results are: First, MES frequency during PFA using CMAC or VLCC is three to 10 times higher compared to applying HPSD RFA. Second, HPSD RFA for AF is associated with a very low number of MES. Third, the specific procedural step that is associated with the highest number of MES is the ablation itself.

### 4.1 MES Formation During Ablation Using Radiofrequency‐ Versus Pulsed Field Ablation

PFA is a cardioselective ablation technique which reduces procedural complexity and duration, and thus appears to have a favorable safety profile [20]. So far, two randomized trials have compared PFA to traditional thermal ablation, and have shown similar acute and long‐term outcomes [22, 23]. Evidence from both trials indicates that PFA is associated with the occurrence of both subclinical and clinical cerebral embolism.

In the ADVENT trial, one transient ischemic attack occurred in the PFA cohort, corresponding to an incidence of 0.3% [22]. In the SINGLE SHOT CHAMPION trial, one stroke was observed in the PFA group, equivalent to approximately 0.95% incidence [23]. In the ADVENT trial, 33 patients had postprocedural MRI, showing SCL in three patients (9%), versus no detected SCL in the thermal ablation group [24]. Additionally, SCL rate may be considerably higher when using different PFA catheters [25], showing SCL rates of up to 85% (VLCC group) and 22% in the CMAC group.

Our results are in line with a recently published study by Shiomi et al. also comparing MES rate between different PFA systems (FARAPULSE (Inc, Menlo Park, CA), VLCC, CMAC). In this trial, embolic burden was also highest during ablation. Ablation using a VLCC catheter had the highest MES counts (858 ± 266), followed by CMAC (472 ± 337). MES detection in FARAPULSE was lowest (61 ± 45) [26]. Notably, 36% of patients treated with VLCC had multiple SCEs/SCLs. Both studies show marked differences between the ablation systems and catheters with underlying causes likely beyond energy modality. We believe these results should have an impact on further catheter and ablation system development.

While MES formation in RFA has been linked to blood coagulation [27], charring [28] and gas bubble formation [29], the mechanism in PFA is less clear. PFA allows variations of several parameters, including amplitude, number of applications, polarity, pulse duration and pauses between trains. All of these settings, in addition to catheter design, electrode size and spacing may influence MES formation. As with RFA, insufficient catheter‐tissue contact may lead to higher exposure of blood within the electrical field causing microthrombi formation. A recent study suggests that even in PFA, significant tissue heating can occur, facilitated by low irrigation rate and an increased number of applications, thereby possibly contributing to MES formation [30].

It should be noted that the MES count during RFA procedures in our study with unilateral detection is among the lowest ever reported. Previous studies have shown that the MES burden during RFA is lower with the use of force sensing catheters, higher ACT values [31], irrigated ablation [12] avoidance of drag ablations [32] and avoidance of left atrial angiography [31]. Additionally, a low number of transseptal punctures may further reduce formation of MES. The use of a HPSD RFA protocol may also contribute to the lower MES number, as the aforementioned studies had higher MES counts while using conventional power settings.

### 4.2 MES Formation During Different Steps of the Ablation Procedure

In this study, the vast majority of MES were detected during the ablation phase. Only a limited number of previously published studies provide detailed information on the timing of MES formation during ablation procedures. Using different energy sources and ablation catheters they showed mixed results.

One study including 53 patients undergoing PVI with RFA using a CoolFlex (St. Jude Medical, St. Paul, MN, USA) or ThermoCool Surround flow (Biosense Webster Inc) catheter using 40 Watts found the highest MES frequency during ablation [31] and atrial angiography. Studies investigating phased RFA (“Pulmonary Vein Ablation Catheter” (PVAC, Medtronic Inc., Minneapolis, Minnesota, USA) also showed MES formation mainly in the ablation period [11, 33, 34].

In contrast to these results, MES were primarily observed after transseptal puncture until first ablation in two studies using either a Celsius Thermocool catheter (Biosense Webster, Diamond Bar, CA, USA) [12] or Thermocool Smarttouch SF catheter (Biosense Webster, Diamond Bar, CA, USA) [35]. In both studies double transseptal puncture was performed and oral anticoagulation was stopped several days before ablation. Also, mean ACT values were lower compared to our study cohort at 210 [12], which may in part explain the differing results.

In a recently published study using the 4th generation cryoballoon, MES were observed frequently during left atrial angiography (7). Additionally, cases with left atrial roof line on top of standard PVI did not show a higher MES burden compared to PVI alone (146, IQR 94–336 vs. 125, IQR 90–175; *p* = 0.13).

In the present study higher numbers of MES during CMAC were also observed during transseptal puncture and catheter introduction. It can be hypothesized that the larger transseptal sheath needed for CMAC, or the catheter design itself may have contributed to these findings. On the other hand, absolute MES differences were small and therefore unlikely to be of clinical relevance.

## 5 Limitations

This prospective study showed significant differences between methods of CA and identified its most dangerous procedural steps. Limitations have to be considered and include the single center design making selection bias possible. Second, the study lacks a bihemispheric MES‐load as unilateral TCD was used in all patients. However, studies suggest no differences in MES detection between right and left middle cerebral artery [8, 36]. Doubling unilateral MES counts to estimate total count seems to be reliable, and is easier to implement, since bilateral TCD is only possible in about 1/3 of patients [8, 37]. Also, heparin was given after femoral access but before the transseptal puncture. This timing may have influenced the embolic burden, particularly during the early stages of the procedure.

Third, this study does not include long‐term outcome but is confined to short‐term clinical outcome with post‐procedural neurological testing performed at the same day/day after the procedure.

Fourth, patients with PFA presented more frequently with persistent atrial fibrillation, which could have contributed to the higher MES burden [38]. However, no additional extrapulmonary vein ablation was performed, and median CHADS‐Vasc‐Score and left atrial scarring did not differ between groups. Moreover, the number of ablation applications did not differ between patients with paroxysmal and persistent AF in the PFA group.

We performed electroanatomical mapping in the CMAC group using a pentaspline catheter after the ablation which might have introduced additional MES via catheter change. Yet, we observed no elevated MES counts after this step. Finally, it should be mentioned that all patients underwent assessment for neurological alterations using a standardized testing procedure, which may have led to an increased detection of symptoms.

## 6 Conclusion

Pulsed field ablation was associated with a higher cerebral MES count than high‐power short‐duration radiofrequency ablation, with marked differences between catheter systems. Transcranial doppler indicates ablation as the most embolic step in both PFA and high‐power short duration ablation.

## Conflicts of Interest

The authors declare no conflicts of interest.

## Data Availability

The authors have nothing to report.
